# Tigecycline-induced sustained severe hypoglycemia: a case report

**DOI:** 10.1186/s40360-019-0321-y

**Published:** 2019-08-19

**Authors:** Yixin Chen, Lin Li, Nan Zhang, Hong Li

**Affiliations:** Department of Endocrinology, Zhejiang University School of Medicine Sir Run Run Shaw Hospital, 3 East Qing Chun Road, Zhejiang, 310016 Hangzhou China

**Keywords:** Tigecycline, Hypoglycemia, Multi-drug resistant organism

## Abstract

**Background:**

Tigecycline, the first glycylcycline-class drug, is a broad-spectrum antibiotic with activity against multi-drug resistant (MDR) organisms. We describe a case of sustained and severe hypoglycemia in a patient treated with tigecycline for pneumonia due to MDR *Klebsiella pneumoniae*.

**Case presentation:**

A 74-year-old man was admitted for treatment of pneumonia. At admission he had prediabetes. In the hospital he developed renal failure. On day 3, the patient experienced severe shortness of breath. He was intubated and transferred to the intensive care unit (ICU) for ventilator support. In the ICU the antibiotic regimen was cefoperazone and sulbactam (1 g every 12 h). Continuous Renal Replacement Therapy was started on that day. Test for anti-neutrophil cytoplasmic antibodies (ANCA) was positive, and so the nephrologist and rheumatologist agreed on a diagnosis of ANCA-associated vasculitis, with renal and pulmonary involvement and acute renal failure. Plasmapheresis, and high-dose methylprednisolone treatment were started on day 6, and there was obvious improvement in the patient’s condition. The steroid regimen was gradually tapered to oral prednisone (35 mg every day) on day 19. Afterwards, the patient’s pneumonia worsened. Sputum culture showed *Klebsiella pneumoniae* sensitive to only tigecycline. Tigecycline treatment (100 mg every 12 h) was administered on day 20. Hypoglycemia started about 37 h after the first dose of tigecycline. Infusion of 50% glucose through the femoral vein was required for over 20 h to maintain normal blood glucose concentrations. Tigecycline was stopped, but the hypoglycemia resolved only after a further 34 h. The insulin and C-peptide levels were found to be markedly elevated during the hypoglycemia. The Naranjo scale score of 7 indicated that the likelihood of tigecycline causing severe hypoglycemia was “probable.”

**Conclusion:**

This is the first report of sustained severe hypoglycemia due to tigecycline. Oversecretion of insulin appears to have been the cause of the hypoglycemia in our patient. The mechanism needs to be investigated.

**Electronic supplementary material:**

The online version of this article (10.1186/s40360-019-0321-y) contains supplementary material, which is available to authorized users.

## Background

Tigecycline is the first glycylcycline-class antibiotic to be available in parenteral form. It has been approved by the US Food and Drugs Administration (FDA) for treatment of complicated skin and intra-abdominal infections and community-acquired bacterial pneumonia. Tigecycline is a broad-spectrum antibiotic, with proven activity against multidrug-resistant (MDR) organisms. In clinical trials the most frequently reported adverse reactions were nausea, vomiting, and diarrhea. Hypoglycemic events were rare (< 2%) [1]; severe hypoglycemia has never been reported.

We describe a case of sustained severe hypoglycemia in a patient treated with tigecycline for pneumonia caused by MDR *Klebsiella pneumoniae*. Our description followed of the case followed the CARE guidelines and the CARE checklist was provided as an additional file (Additional file 1).

## Case presentation

A 78-year-old man with complaints of cough and trouble breathing for 1 month was admitted to the ward of the Respiratory Medicine Department of Sir Run Run Shaw Hospital on the 12th January 2018. He had been seen earlier in the outpatient clinic on 23th December 2017. At that visit a chest computed tomography (CT) scan had demonstrated pneumonia; echocardiogram had shown thickening of the left ventricular wall, with ejection fraction of 65.8%; and the serum creatinine had been 76 μmol/L, indicating normal renal function. He had been treated with levofloxacin (0.5 g every day) for 1 week in the outpatient department. As there was no improvement, levofloxacin was substituted with moxifloxacin (0.4 g every day) and he was advised hospitalization for further therapy.

At admission, the patient was conscious and well oriented. A digital electronic sphygmomanometer showed blood pressure of 144/74 mmHg and heart rate of 96 beats/min. The respiratory rate was 18 breaths/min and the temperature was 36.3 °C. There were no rales or wheezes heard in the chest. Mild ankle edema was present. Physical examination was otherwise normal.

The patient had history of hypertension for over 10 years and was on nifidipine, with good control of blood pressure. He had no history of diabetes. However glycated hemoglobin (HbA1c) was 6.2%, suggesting impaired glucose metabolism. His gallbladder had been removed 30 years ago. He was married and had two children. All family members were in good health.

After admission, antibiotic treatment was started with piperacillin and tazobactam (4.5 g every 8 h). His serum creatinine level rose sharply, and on the day 2 of admission it was 506 μmol/L. It continued to rise over the following days. A nephrology consultant suggested two possibilities: allergic interstitial nephritis and renal vasculitis, and advised tests for autoantibodies. Blood was drawn and sent to the laboratory.

On day 3, however, the patient experienced severe shortness of breath. He was intubated and transferred to the intensive care unit (ICU) for ventilator support. In the ICU the antibiotic regimen was changed to cefoperazone and sulbactam (1 g every 12 h). Continuous Renal Replacement Therapy (CRRT) was started on that day.

On day 5, test for anti-neutrophil cytoplasmic antibodies (ANCA) was positive, and so the nephrologist and rheumatologist agreed on a diagnosis of ANCA-associated vasculitis, with renal and pulmonary involvement and acute renal failure.

Plasmapheresis, and intravenous methylprednisolone treatment (500 mg every day for 3 days) were started on day 6, and there was obvious improvement in the patient’s condition. On day 7, he was taken off the ventilator and transferred to the nephrology department. Hemodialysis and antibiotic therapy were continued. The steroid regimen was gradually tapered to oral prednisone (35 mg every day) on day 19.

**Fig. 1:**
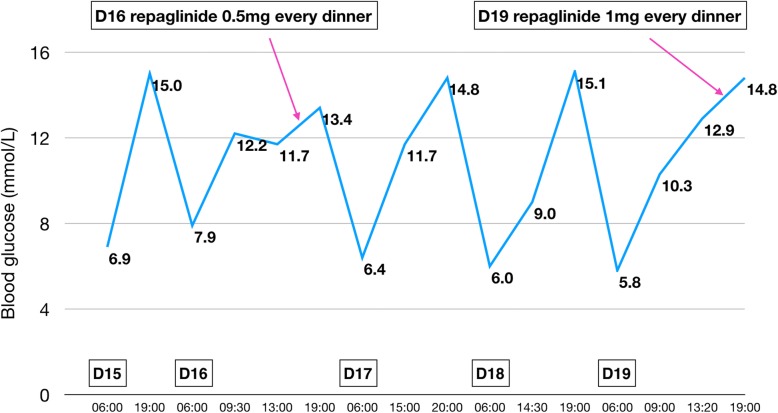
The patient’s glycemic profile on days 15–19. Repaglinide was administered (0.5 mg with dinner) on day 16. The dose was doubled on day 19

Over the next few days the patient’s pneumonia worsened. He complained of increasing chest tightness, and there was progressive increase in the levels of inflammatory markers C-reactive protein and procalcitonin. Chest CT revealed increase in lung consolidation and inflammation. On day 15 cefoperazone and sulbactam were substituted with meropenem (0.5 g every 12 h). By this time his blood glucose had increased as a result of the methylprednisolone treatment. His postprandial glucose concentration after dinner was 15 mmol/L. He was started on repaglinide (0.5 mg with dinner) on day 16. On day 18 the results of sputum cultures showed infection with *Candida albicans* and multi-resistant *Klebsiella pneumoniae* sensitive only to tigecycline. Voriconazole (400 mg every 12 h) was started on day 18, and meropenem was replaced with off-label tigecycline (100 mg every 12 h) on day 20. Meanwhile, on day 19, the dose of repaglinide was increased to 1 mg with dinner (Fig. 1). The patient’s chest tightness was partly relieved, and serum procalcitonin decreased sharply, indicating response to the new antibiotic.

**Fig. 2:**
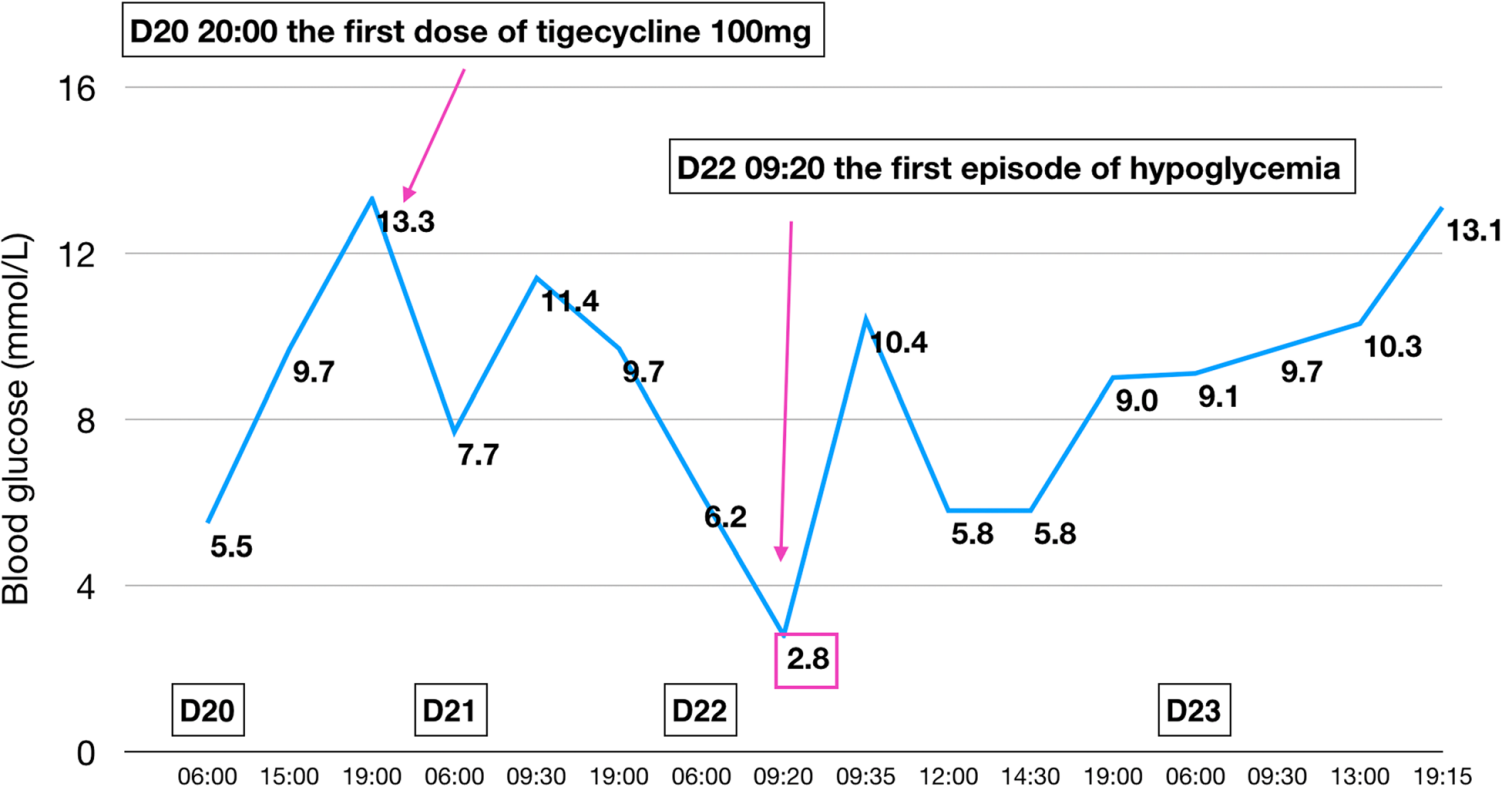
The patient’s glycemic profile on days 20–23. Intravenous tigecycline (100 mg every 12 h) was started on day 20. The first hypoglycemic attack was on day 22

**Fig. 3:**
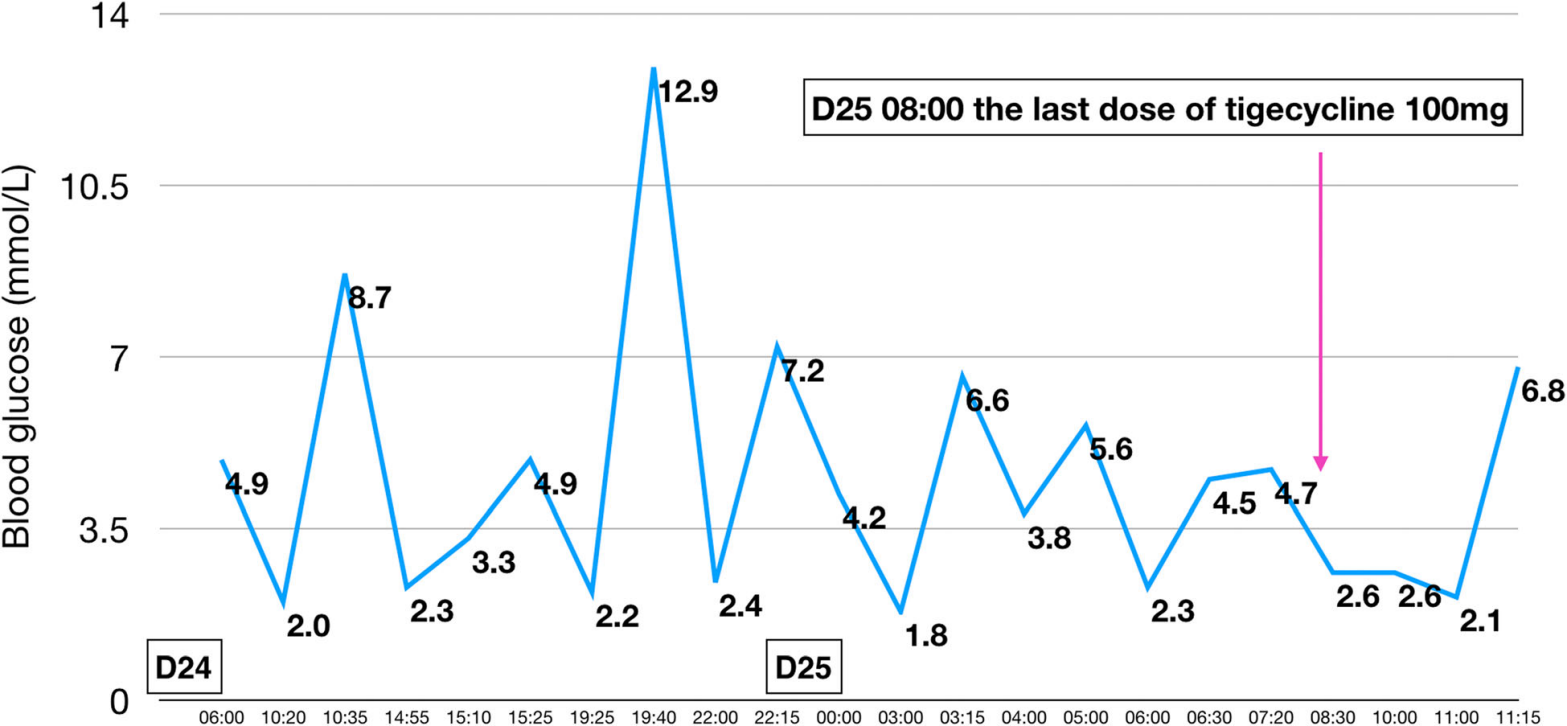
The patient’s glycemic profile on days 24–25. The patient had four hypoglycemic episodes on day 24. A sustained and more severe hypoglycemic attack occurred on day 25. The last dose of tigecycline was administered at 8 AM on day 25

**Fig. 4:**
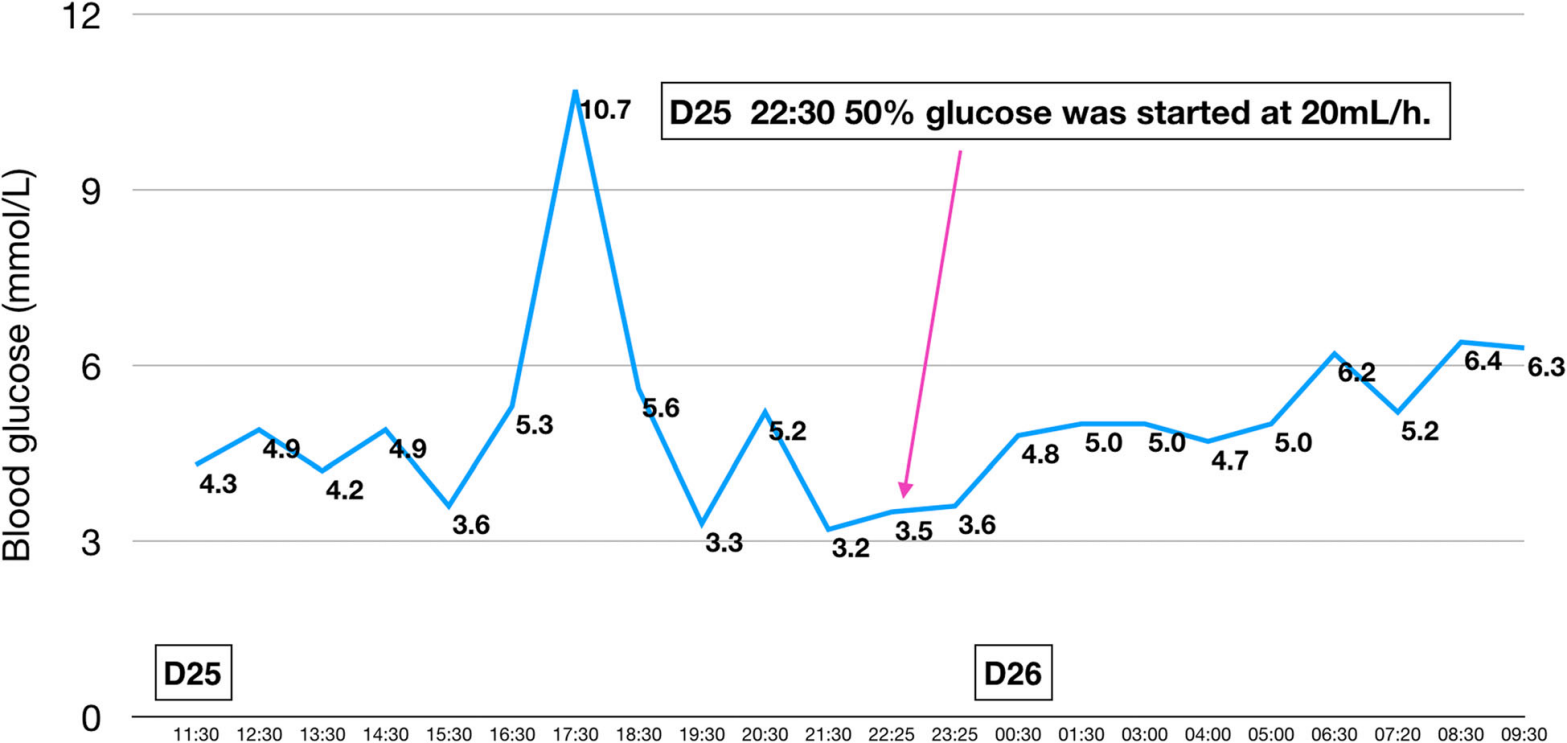
The patient’s glycemic profile on days 25–26. The hypoglycemia continued. Continuous 50% glucose infusion through the femoral vein was started on the night of day 25

**Fig. 5:**
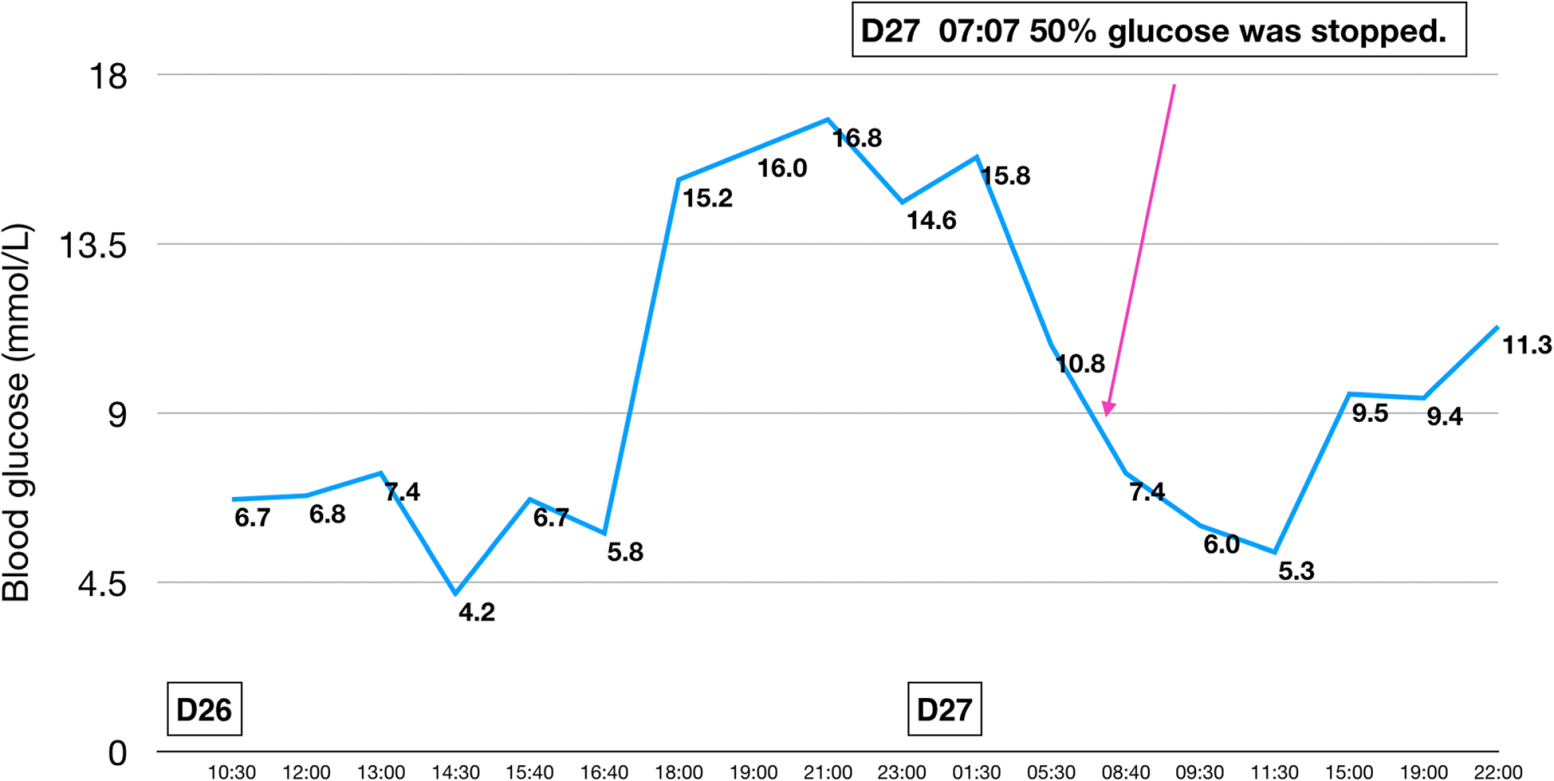
The patient’s glycemic profile on days 26–27. The blood glucose showed an obviously rise at 6 PM on day 26 and remained above 10 mmol/L from then on, indicating probable resolution of the hypoglycemia. The 50% glucose infusion was discontinued on day 27

On day 22 the patient experienced a typical hypoglycemic attack after breakfast, manifesting with palpitations, tremor, sweating, and hunger. Glucometer showed blood glucose to be 2.8 mmol/L. The symptoms were promptly relieved with 20 g of oral glucose, and the blood glucose rose to 10.4 mmol/L. No hypoglycemic event occurred the next day (Fig. 2). On day 24, however, the patient suffered another hypoglycemic attack. This time, it was severe and sustained. Glucometer showed blood glucose to be 2.0 mmol/L. The patient was confused and irritable. He was administered 40 mL 50% glucose intravenously, following which blood glucose rose to 8.7 mmol/L. However, three further hypoglycemic episodes occurred on the same day. Each time the blood glucose concentration was restored to normal with intravenous 50% glucose. Repaglinide was stopped on day 24. The nephrologist consulted us on day 25 to discuss the course of further treatment. After evaluation of the patient’s drug history and clinical manifestations, we decided that tigecycline was the likely cause of the hypoglycemia and stopped the drug (Fig. 3). However, the hypoglycemia persisted. Oral glucose, repeated doses of intravenous 50% glucose solution, and infusion with 16.7% glucose (50 mL 10% glucose + 10 mL 50% glucose) solution could not maintain normal blood glucose concentrations. Therefore, femoral vein cannulation was performed and 50% glucose solution was infused (Fig. 4). With this, the blood glucose concentration was maintained at 3.6–7.4 mmol/L over the next 20 h. At 6 PM on day 26 the blood glucose showed an obvious rise and remained above 10 mmol/L through the rest of the night, indicating that the hypoglycemia had probably been corrected. Intravenous 50% glucose infusion was stopped on day 27 (Fig. 5). There were no further episodes of hypoglycemia.

During the episodes of hypoglycemia, insulin and C-peptide levels were also measured along with blood glucose; both were found to be elevated whenever hypoglycemia occurred. Figures 1, 2, 3, 4, 5 show the patient’s glycemic profile from day 15 to day 27. Table 1 lists the pertinent laboratory values.

**Table 1:** Results of Laboratory Tests during Hospital Stay

Laboratory test (normal value)	Day 25	Day 26	Day 28
Glucose (4.16–5.83 mmol/L)	2.87	5.06	4.06
Insulin (1.90–23.00 μIU/mL)	33.07	30.99	3.7
C-peptide (250–600 pmol/L)	> 13333	> 13333	2189
Serum cortisol at 8 AM (6.70–22.60 μg/dL)		6.44	
Serum ACTH at 8 AM (10–80 ng/L)		5	
TSH (0.35–4.94 mIU/L)		2.65	
Insulin autoantibody (negative)		Negative	

*ACTH* Adrenocorticotropic hormone, *TSH* Thyroid-stimulating hormone

On day 28, polymyxin B and imipenem were started for treatment of the pneumonia. However, the patient’s condition deteriorated and he died shortly after.

## Discussion and conclusion

Hypoglycemic attacks are not a common adverse reaction in patients receiving tigecycline: only 11 cases have been reported between 2004 and 2009. In 2014, in a phase III study comparing tigecycline and ertapenem in diabetic foot patients with and without osteomyelitis, 34 of 477 patients had hypoglycemic attacks in the tigecycline group [2]. The attacks were much more common in diabetic patients who were also taking glucose-lowering treatment.

Our patient was unusual in that he suffered prolonged and severe hypoglycemia; such a reaction has not been reported before. The first episode occurred about 37 h after the first dose of tigecycline. Two days later, the hypoglycemia recurred, and this time it was sustained. Tigecycline was stopped, but the hypoglycemia was not relieved until 34 h after the last dose of tigecycline. According to the prescription information from the FDA [1], the single-dose half-life of 100 mg of tigecycline is 27 h. The duration of the hypoglycemia in our patient was consistent with the pharmacokinetics of tigecycline.

Our patient had received repaglinide before the hypoglycemic event. However, this drug cannot cause such sustained hypoglycemia as it has a short half-life of only about 1 h [3]. In fact, diabetic patients usually need multiple doses of repaglinide each day to ensure blood glucose control. Moreover, 90% of the metabolites of repaglinide are excreted through the bile, and so the clearance rate is unaffected by renal failure.

Interestingly, in our patient, insulin and C-peptide levels were markedly elevated even though blood glucose was low, indicating that overproduction of insulin was the main cause of the hypoglycemia. Three days after tigecycline was stopped there was a sharp decline in the insulin and C-peptide levels. Thus, it appears that tigecycline stimulates the secretion of insulin. However, the mechanism of action is not yet clear. In our patient, impaired renal function would have resulted in delayed clearance of insulin and thus led to prolongation of hypoglycemia.

**Fig. 6:**
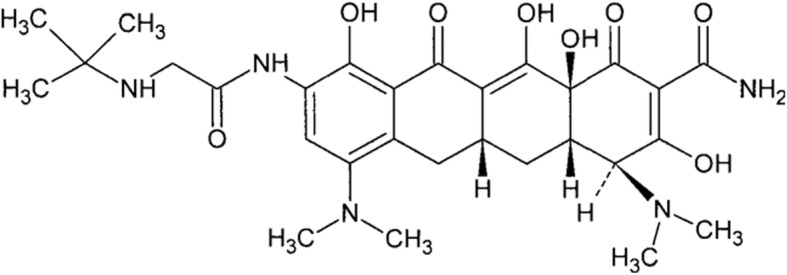
Chemical structure of tigecycline. Tigecycline contains no sulfhydryl group

Our patient had autoimmune disease, as indicated by the positive test for ANCA. This led us to consider insulin autoimmune syndrome as a possible cause of the hypoglycemia. However, this possibility was ruled out by the negative insulin autoantibody test and the fact that tigecycline contains no sulfhydryl group (Fig. 6).

The Naranjo Adverse Drug Reaction Probability Scale [4] showed a probable relationship (score of 7) between the patient’s severe hypoglycemia and tigecycline treatment (Table 2). Because of ethical considerations we could not restart treatment with tigecycline despite its antibiotic efficacy in this patient.

**Table 2:** The Patient’s Scores for the Naranjo Adverse Drug Reaction Probability Scale Questions

To assess the adverse drug reaction, please answer the following questionnaire and give the pertinent score.				
	Yes	No	Do not know	Score
I. Are there previous conclusive reports on this reaction?	+ 1	0	0	+ 1
2. Did the adverse event appear after the suspected drug was administered?	+ 2	−1	0	+ 2
3. Did the adverse reaction improve when the drug was discontinued or a specific antagonist was administered?	+ 1	0	0	+ 1
4. Did the adverse reaction reappear when the drug was re-administered?	+ 2	−1	0	0
5. Are there alternative causes (other than the drug) that could on their own have caused the reaction?	−1	+ 2	0	+ 2
6. Did the reaction reappear when a placebo was given?	−1	+ 1	0	0
7. Was the drug detected in the blood (or other fluids) in concentrations known to be toxic?	+ 1	0	0	0
8. Was the reaction more severe when the dose was increased, or less severe when the dose was decreased?	+ 1	0	0	0
9. Did the patient have a similar reaction to the same or similar drugs in any previous exposure?	+ 1	0	0	0
10. Was the adverse event confirmed by any objective evidence?	+ 1	0	0	+ 1
			Total score	7

To summarize, we report a patient who suffered prolonged and severe hypoglycemia while on treatment with tigecycline. The hypoglycemia resolved only 34 h after stoppage of the drug. The Naranjo scale score of 7 indicated that a “probable” relationship exists between tigecycline administration and the severe hypoglycemia. Insulin and C-peptide levels showed a close inverse relationship with blood glucose, indicating that oversecretion of insulin was the likely cause of the prolonged hypoglycemia. Further investigations are needed to clarify the underlying mechanisms. To our knowledge, this is the first report of sustained severe hypoglycemia due to tigecycline.

## Additional file

Additional file 1: The CARE checklist. (XLSX 11 kb)

## Data Availability

The dataset analyzed during the current study are available in the Mendeley. (10.17632/3ym3x4ckyn.1).

## Abbreviations

ANCA: Anti-neutrophil cytoplasmic antibodies
CT: Computed tomography
FDA: Food and Drugs Administration
HbA1c: Glycated hemoglobin
ICU: Intensive care unit
MDR: Multi-drug resistant
